# Association between changes in lean mass, muscle strength, endurance, and power following resistance or concurrent training with differing high protein diets in resistance-trained young males

**DOI:** 10.3389/fnut.2024.1439037

**Published:** 2024-08-14

**Authors:** Reza Bagheri, Zohreh Karimi, Donny M. Camera, David Scott, Mahdi Ziaee Bashirzad, Ramin Sadeghi, Mehdi Kargarfard, Fred Dutheil

**Affiliations:** ^1^Department of Exercise Physiology, Faculty of Sport Sciences, University of Isfahan, Isfahan, Iran; ^2^Department of Physical Education and Sport Sciences, Islamic Azad University of Central Tehran Branch, Tehran, Iran; ^3^Department of Health and Biostatistics, Swinburne University, Melbourne, VIC, Australia; ^4^Institute for Physical Activity and Nutrition (IPAN), Deakin University, Geelong, VIC, Australia; ^5^School of Clinical Sciences at Monash Health, Monash University, Clayton, VIC, Australia; ^6^Department of Sport Science, Islamic Azad University, Bojnurd Branch, Bojnurd, Iran; ^7^Nuclear Medicine Research Center, Mashhad University of Medical Sciences, Mashhad, Iran; ^8^Université Clermont Auvergne, CNRS, LaPSCo, Physiological and Psychosocial Stress, CHU Clermont-Ferrand, University Hospital of Clermont-Ferrand, Preventive and Occupational Medicine, Witty Fit, Clermont-Ferrand, France

**Keywords:** exercise training, body composition, nutrition, nutritional supplements, muscular adaptations

## Abstract

**Background:**

We assessed the relationship of changes in upper and lower body lean mass with muscle strength, endurance and power responses following two high protein diets (1.6 or 3.2 g^.^kg^-1.^d^−1^) during 16 weeks of either concurrent training (CT) or resistance training (RT) in resistance-trained young males.

**Methods:**

Forty-eight resistance-trained young males (age: 26 ± 6 yr., body mass index: 25.6 ± 2.9 kg.m^−2^) performed 16 weeks (four sessions·wk.^−1^) of CT or RT with either 1.6 g^.^kg^-1.^d^−1^ protein (CT + 1.6; *n* = 12; RT + 1.6; *n* = 12) or 3.2 g^.^kg^-1.^d^−1^ protein (CT + 3.2; *n* = 12; RT + 3.2; *n* = 12). Relationships between upper (left arm + right arm + trunk lean mass) and lower body (left leg + right leg lean mass) lean mass changes with changes in muscle performance were assessed using Pearson’s correlation coefficients.

**Results:**

For upper body, non-significant weak positive relationships were observed between change in upper body lean mass and change in pull-up (*r* = 0.183, *p* = 0.234), absolute chest press strength (*r* = 0.159, *p* = 0.302), chest press endurance (*r* = 0.041, *p* = 0.792), and relative chest press strength (*r* = 0.097, *p* = 0.529) while non-significant weak negative relationships were observed for changes in absolute upper body power (*r* = −0.236, *p* = 0.123) and relative upper body power (*r* = −0.203, *p* = 0.185). For lower body, non-significant weak positive relationships were observed between the change in lower body lean mass with change in vertical jump (*r* = 0.145, *p* = 0.346), absolute lower body power (*r* = 0.109, *p* = 0.480), absolute leg press strength (*r* = 0.073, *p* = 0.638), leg press endurance (*r <* 0.001, *p* = 0.998), relative leg press strength (*r* = 0.089, *p* = 0.564), and relative lower body power (*r* = 0.150, *p* = 0.332).

**Conclusion:**

Changes in muscle strength, endurance and power adaptation responses following 16 weeks of either CT or RT with different high protein intakes were not associated with changes in lean mass in resistance-trained young males. These findings indicate that muscle hypertrophy has a small, or negligible, contributory role in promoting functional adaptations with RT or CT, at least over a 16-week period.

## Introduction

Resistance training (RT) is considered the most effective approach to promote anabolic-related adaptations, including increases in muscle strength, and power in trained adults (1). On the other hand, endurance training (ET) may lead to improvements in VO_2max_, resulting in enhanced cardiovascular health and function, and an increase in skeletal muscle oxidative capacity (2). Considering the distinct training adaptations of ET and RT, which can be influenced by various factors including training type, intensity, and volume, integrating both modalities into a cohesive training regimen to maximize concurrent anabolic, metabolic, and oxidative adaptation responses is often required for exercise/sport performance and overall health and wellbeing. Concurrent training (CT) involves the integration of RT and ET into a unified training protocol (3) and has been shown to augment muscle strength, anaerobic power, aerobic capacity, and maximum velocity contractions (4–8). Despite the favorable adaptations observed with CT, some studies have demonstrated diminished enhancements in muscle strength, power, and hypertrophy when compared to RT conducted independently, which is often referred to as the “interference effect” (9–14). The literature presents contradictory findings about dampened anabolic training responses within this paradigm, which may be influenced by factors such as participants’ training experience, the order in which training sessions are conducted, and the specific forms of exercise implemented (15–18).

Various proposed methods for overcoming the interference effect have focused on implementing longer periods of recovery (i.e., 6–24 h) between training sessions, substituting cycling for running as ET, and integrating post-exercise dietary strategies (19). Various studies have shown that protein ingestion, either via food or supplements, in combination with RT, leads to improved muscle adaptations such as increased strength, lean mass, or power (1, 20–27). Greater gains in lean mass (3.2 vs. 2.2 g^.^kg^-1.^d^−1^) have been reported with higher protein intake (~2.2–2.4 g^.^kg^-1.^d^−1^) compared to ~1.0 g^.^kg^-1.^d^−1^ following three, but not six, months of CT in recreationally trained males (28). This finding suggests higher daily protein availability can significantly impact adaptation responses with CT. Moreover, results of a recent systematic analysis support the use of protein supplementation to improve the growth of skeletal muscle mass and increase strength and power when combined with CT. Specifically, consuming approximately 0.49 g/kg after exercise is believed to optimize the rate of myofibrillar protein synthesis in this particular context (29). Morton and colleagues conducted a systematic review and meta-analysis, which resulted in a recommendation. A protein intake of 1.6 g^.^kg^-1.^d^−1^ was enough to provide the maximum increase in fat-free mass (FFM) after RT (26). On the other hand, others have suggested that trained individuals should consume about 2 g^.^kg^-1.^d^−1^ to meet their daily requirements (20). It is important to note that these recommended daily protein amounts are derived only from research that includes RT. Due to the larger training volumes associated with CT compared to single-mode exercise training, it is probable that the necessary dietary protein intake is greater for CT (3). Regardless, increasing muscle/lean mass is highly important for promoting physical performance and health among all populations (30) with the quantity of lean mass shown to be associated with performance indicators, including aerobic and anaerobic capacities (31–34). Thus, dietary strategies that can help facilitate increases in muscle mass, strength and functional adaptations with CT represents a highly important consideration for individuals undertaking this form of exercise training.

There is a paucity of information regarding whether potential associations between changes in muscle mass and strength exist with CT. Moreover, much less is known about the correlation between increases in lean mass resulting from RT or CT following high-protein diets and muscle performance in resistance-trained males. Dietary protein supplementation has been shown to significantly enhance changes in muscle strength and size with RT in healthy adults (26) and thus represents an important variable in maximizing skeletal muscle adaptation responses to exercise training. We have previously reported that RT and CT in combination with higher-protein diets (i.e., 1.6 [1.6] or 3.2 [3.2] g.kg^−1^.d^−1^) similarly improved lean mass and selected muscle performance measures (35). In light of this, the current study aimed to explore the relationship between changes in lean mass and muscle strength and power following 16 weeks of RT and CT in conjunction with consumption of with 1.6 or 3.2 g.kg^−1^.d^−1^ of protein in resistance-trained young males. It was primarily hypothesized that post-intervention increases in muscle growth responses will correlate with changes in muscle strength. Additionally, as there were no differences between the magnitude of gains in lean mass among RT and CT groups in our previous study findings, it was also hypothesized that there will no differences between RT and CT groups regarding the association between lean mass and muscle strength, endurance, and power adaptations.

## Methods

### Participants

The current investigation recruited 48 resistance-trained young males. These individuals were between the ages of 18 and 36 and were recruited via the use of advertising on various social media platforms. The research and testing protocols were communicated to interested participants via telephone or in-person sessions held at nearby fitness facilities. Participants were instructed to complete a health and fitness history questionnaire, providing information about their previous training background, specifically engaging in three sessions per week for a minimum of 1 year of RT experience (with three to four sessions per week). Additionally, participants were required to report sleeping for a duration of seven to 8 h within a 24-h day, abstaining from the use of steroids or any illegal substances known to enhance muscle size for the past year, consuming less than ~1.6 g^.^kg^-1.^d^−1^, and being free from any musculoskeletal disorders. Participants who met the aforementioned criteria supplied a written and verbal agreement to participate in the study. Furthermore, as part of the permitting process, participants were provided with a medical history questionnaire and revisited the research site to complete the study procedures. The procedure underwent a thorough evaluation by the Institutional Human Subject Committee and the Ethics Committee of the University of Isfahan (IR.UI.REC.1400.098) and was conducted strictly adhering to the principles outlined in the Declaration of Helsinki. The present research has been duly filed with the Iranian Registry of Clinical Trials with the registration number IRCT20191204045612N2.

### Study design

Following the collection of baseline measures, as detailed in the following section, participants were familiarized with the research tests and procedures. Subsequently, they were randomized to one of four groups using the use of an online resource:^1^ CT + 1.6 g protein^.^kg^-1.^d^−1^ (CT + 1.6; *n* = 12), CT + 3.2 g protein^.^kg^-1.^d^−1^ of protein (CT + 3.2; *n* = 12), RT + 1.6 g protein^.^kg^-1.^d^−1^ (RT + 1.6; *n* = 12) or RT + 3.2 g protein^.^kg^-1.^d^−1^ (RT + 3.2; *n* = 12). The initial planned duration of this research was 6 months; however, in response to the global COVID-19 pandemic, we made a voluntary decision to conclude the study after 16 weeks. Consequently, data were gathered at the first assessment and the 18th week, specifically 2 weeks after the implementation of the training intervention, at the same time of day, with a time difference of ±1 h. After completing these assessments, study participants engaged in an initial consultation with the research’s dietician. This meeting served as an opportunity to discuss their individual dietary preferences and establish specific protein and calorie intake goals in preparation for the commencement of their respective training programs. To assess sleep quality and health status, the Pittsburgh Sleep Quality Index (PSQI) and the General Health Questionnaire-28 (GHQ-28) were used, respectively (36).

### Anthropometry and body composition

Participants were asked to report to the laboratory after an overnight fast, with a 24-h dietary recall collected before testing. Participants were instructed to void completely within 30 min of the test to minimize hydration status errors and advised to refrain from caffeinated beverages, alcohol, and other diuretics 12 h before measurements. Participants’ body mass and height were measured with a digital scale (Lumbar, China) to the nearest 0.1 kg and a stadiometer (Race industrialization, China) to the nearest 0.1 cm. Total lean mass was assessed using whole-body dual-energy x-ray absorptiometry (DXA; Hologic, Discovery, Wi [S/N 93045 M]). For DXA measurements, previous test re-test reliability in our laboratory are as follows: BFP intraclass correlation coefficient (ICC) = 0.998; coefficient of variation (CV): <1%; lean mass: ICC = 1.00; CV: <1%. All DXA scans were conducted as described previously (37). In the present study, we assessed the relationships between upper (left arm + right arm + trunk lean mass) and lower body (left leg + right leg lean mass) lean mass and muscle performance changes.

### Resistance training

The participants in the two RT groups engaged in a structured exercise regimen consisting of four weekly sessions as described previously. Prior to each RT session, participants performed 10 min of general (5 min slow running on a treadmill; 3–5 km speed, or elliptical; with 5–10 level) and specific warm-up activities (5 min, e.g., medicine ball twist 1 × 10, medicine ball wood chops 1 × 10, straddled toe touch 2 × 5, dynamic quadriceps stretch 1 × 5, medicine ball squat 1 × 5–8). Participants then completed an upper-body RT program consisting of seven exercises (chest press, lateral pulldown, standing barbell shoulder press, standing shoulder shrugs, bicep curl, triceps press down, and abdominal crunch) 2x/wk. and six exercises of lower-body RT program (45-degree leg press, back squats, seated leg curl, Barbell hip thrusts, back extension, and calf raises) performed for 2x/wk. Participants performed 3 sets of 12 repetitions with 75% of 1-RM for weeks 1–4, 3 sets of 10 repetitions with 80% of 1-RM for weeks 5–8, 4 sets of 8 repetitions with 85% of 1-RM for weeks 9–12, and 4 sets of 6 repetitions with 90% of 1-RM for weeks 13–16. Rest intervals between exercises and sets lasted no longer than 2 min (38). The periodized RT program was based on our previous work (38) and following recommendations by the National Strength and Conditioning Association (39). Verbal encouragement and comments were provided to the participants both during and after each set. The training data for each participant was recorded, ensuring that the training intensity was optimized throughout each session and that participants effectively adopted progressive overload in a personalized manner. In addition, study personnel supervised all training throughout the study. A detailed outline can be found in Supplementary Table 1.

### Concurrent training

The participants in both CT groups engaged in a total of four sessions each week, namely on Saturday, Monday, Wednesday, and Thursday. Each session consisted of RT done at the start, followed by ET, as per the prescribed exercise order sequence (40) to minimize possible interferences in muscle anabolism. Prior to each CT session, participants performed 10 min of general (5 min slow running on a treadmill; 3–5 km speed, or elliptical; with 5–10 level) and specific warm-up activities (5 min, e.g., medicine ball twist 1 × 10, medicine ball wood chops 1 × 10, straddled toe touch 2 × 5, dynamic quadriceps stretch 1 × 5, medicine ball squat 1 × 5–8). The participants then engaged in the same RT program as previously stated. Immediately following the completion of RT, participants then performed endurance cycle training on ergometers that consisted of a mixture of hill simulation rides of varying intensities (25–110 of maximum aerobic power [MAP]), moderate-intensity continuous training at 50% MAP, moderate-intensity continuous training (MICT) at 70% MAP, and high-intensity interval training (HIIT) at 100% MAP. Moderate-intensity intervals were separated by a 60-s recovery period at ~40% MAP to establish a 2.5:1 or 5:1 work-to-rest ratio. High-intensity intervals were separated by 20- to 60-s recovery periods, completed at ~40% MAP, to establish a 1:5, 1:2, or 1:1 work-to-rest ratio. All cycling sessions were preceded by 3–5 min of cycling at ≤50 W. Progressive overload was applied by manipulating the number of intervals and relative intensity of load throughout. A detailed outline can be found in Supplementary Tables 2A,B.

### Training volume

RT volume was calculated using the following formula in each session and was reported weekly (41):

1. RT volume = [repetitions (n) × sets (n) × load or selected weight (kg)].
2. ET volume was calculated using the following formula: Total ET volume: [work + rest].
3. Work: [Intensity × maximum aerobic power (MAP) × (set × duration [as noted in training protocol] × 0.06)].
4. Rest: [Intensity × MAP × (set × duration [as noted in training protocol] × 0.06)].
5. Intensity: percent of MAP; Set: number of repetitions of each session; Duration: spent time (minutes); 0.06: Convert watts to kilojoules.

### Maximal strength testing

Maximal strength was determined (first upper and then lower limb) using 1-RM for chest press and plate-loaded leg press in the morning. This testing (1-RM) also was performed to determine training intensity for RT protocols. Participants performed a general 10 min warm-up (5 min slow running on a treadmill; 3–5 km speed, or elliptical; with 5–10 level) and specific warm-up activities (5 min, e.g., medicine ball twist 1 × 10, medicine ball wood chops 1 × 10, straddled toe touch 2 × 5, dynamic quadriceps stretch 1 × 5, medicine ball squat 1 × 5–8) before the test. The participants then performed two attempts, recording their highest lifted weight and number of repetitions. The number of repetitions to fatigue did not exceed 10. Participants were allowed 3 to 5 min rest periods between attempts, and there was no arousing stimulus during testing. After the testing session, participant’s maximal strength was predicted using the formula: 1-RM = weight / (1.0278–0.0278 × reps) (19). Chest and leg press exercises were used as upper and lower body strength measures, and 1-RM was used to determine individualized RT prescription.

### Muscle endurance

The participants rested for 5 min after the 1-RM testing prior to completing the muscle endurance test (first upper and then lower limb) in the morning (9:00–10 a.m.). Participants were instructed to perform leg- and chest press exercises at 75% of the 1-RM to test muscle endurance, denoted as the number of successful repetitions completed prior to technical failure (37).

### Muscle power

Upper- and lower-body anaerobic power was assessed via Monark Wingate cycle ergometry (Monark model 894e, Vansbro, Sweden) as previously described in the evening (8, 42). Briefly, participants were acquainted with the test and instructed to stay seated in the saddle for the test duration. Participants cycled or cranked against a pre-determined resistance (7.5% of the body mass for the lower body test and 5.5% for the upper body test) as fast as possible for 30 s. Participants were verbally encouraged to pedal as hard and fast as possible throughout the whole 30-s test. There was a time gap of roughly 1 h between the upper and lower tests, with the upper test being conducted first. Therefore, we can be certain that fatigue did not impact the performance of the alternative limb. Peak power output was documented in real-time during the test using Monark Anaerobic test software (3.3.0.0).

### Muscle performance and power testing

Maximal vertical jump height and total pull-ups (1 set) were assessed. Each participant generally performed the following warm-up: a 5-min run/bike on a treadmill or cycle ergometer at a self-directed leisurely pace followed by a dynamic warm-up consisting of 10 yards each of high knees, butt kicks, side shuffles, and karaoke running drill, and finally 10 pushups and 10 bodyweight squats. Participants then rested for 2–3 min before commencing the muscle strength and power tests. Subsequently, the following tests were performed in the order given: vertical jump—highest value with a maximum number of three attempts; pull-ups—highest repetitions with a maximum number of three attempts. For both tests, there was a rest interval of approximately 60–180 s.

### Diet

The study participants were instructed to record their food intake for a total of six consecutive 24-h periods. These periods included four weekdays that were not consecutive and two non-consecutive weekend days. The purpose of this data collection was to assess the participants’ typical protein intake patterns. To assist in achieving their targeted protein intake (i.e., 1.6 or 3.2 g^.^kg^-1.^d^−1^), participants consumed a 40 g of isolated whey protein (Wisser nutrition, Iran) beverage upon cessation of every training session that comprised the following nutrition profile per scoop (28 g): calories,110; total fat, 0.5 g; saturated and trans-fat, sugars and dietary fiber, 0 g; sodium, 50 mg; potassium, 112 mg; total carbohydrate, 2 g; protein, 24 g. The remaining amounts of protein were received from dietary sources, and the habitual intake of protein remained consistent across all groups during the intervention. The decision to include the protein group with a daily intake of 1.6 g^.^kg^-1.^d^−1^ was based on findings by Morton et al. (26) that reported this quantity of protein intake can optimize improvements in fat-free mass after RT (26). To create a clear disparity between dietary protein interventions, we chose to double the 1.6 g^.^kg^-1.^d^−1^ amount for the comparison high protein group (i.e., 3.2 g^.^kg^-1.^d^−1^) while also ensuring this amount can be safely tolerated. In support, previous work by Antonio and colleagues demonstrated this amount (~2.51–3.32 g^.^kg^-1.^d^−1^) to exert no harmful effects on liver and kidney function markers (43).

The participants engaged in regular consultations with a certified dietitian every fortnight. During these consultations, they received instructions on how to meet their protein and energy requirements. Specifically, they were advised to distribute their protein intake throughout the day across 4–7 meals, with each meal containing 20–40 g of protein. This approach aimed to optimize muscle protein synthesis (MPS) (44, 45). The research included monitoring the macronutrient composition, with particular emphasis on total energy intake (TEI) and protein intake. It has been recommended that individuals maintain their carbohydrate and fat intake within the Acceptable Macronutrient Distribution Range, which suggests a range of 45–65% of total energy intake for carbohydrates and 20–35% of total energy intake for fats. The participants were instructed to maintain a state of positive energy balance to mitigate any possible disruptions to anabolic adaptations caused by energetic stress (46, 47). Food records were kept daily by participants throughout the study using mobile phone applications Easy Diet Diary (Xyris Software Pty Ltd., AUS, for those with iPhones, Apple Inc., United States; *n* = 18) and My Fitness Pal (MyFitnessPal Inc., United States) for those with Android-based devices; *n* = 26. All dietary intake data were analyzed using (Diet Analysis Plus, version 10; Cengage) to ensure the same food database was used for all analyses.

### Statistical analysis

As this study represents a secondary analysis to the original primary aim of comparing muscle effects of different training and protein supplementation protocols (35), no specific sample size calculations were performed for the current work. Nevertheless, prior research on the topic utilized sample sizes that were approximately comparable to the sample size used in this current study (33, 34). The normality of the distribution of all variables was evaluated before performing statistical analyses using the Shapiro–Wilk test; there were no missing values at any time point. Baseline characteristics (at PRE) between groups were reported using mean (SD). Effects of training and nutritional interventions on dependent variables were analyzed using a two × four analysis of variance (ANOVA) with repeated measures (time [pre-test vs. post-test] × group [CT + 1.6 vs. CT + 3.2 vs. RT + 1.6 vs. RT + 3.2]) to determine the differences between the treatments over time. When the group-by-time interaction was significant, we used Sidak *post-hoc* analysis to determine between-group differences. Pearson’s simple linear regressions were performed with a 95% confidence interval (CI) as well as Pearson correlation coefficients. Values between 0 and 0.3 (0 and − 0.3) indicate a weak positive (negative) linear relationship through a shaky linear rule. Values between 0.3 and 0.7 (−0.3 and − 0.7) indicate a moderate positive (negative). Values between 0.7 and 1.0 (−0.7 and − 1.0) indicate a strong positive (negative) (48). Figures with only one curve indicates that it adequately fits all the data sets. All analyses and figure production were performed using GraphPad Prism (version 8.4.3).

## Results

### Participant characteristics

A total of 112 individuals underwent assessment to determine their eligibility. Twenty-eight of them failed to satisfy the established criteria for inclusion, while 36 individuals expressed a lack of interest in participating after the first interview (Supplementary Figure 1). One participant from each group withdrew from the research, citing reasons such as scheduling constraints, lack of interest, COVID-19, or musculoskeletal injury. There were no statistically significant differences seen between the groups in terms of baseline characteristics (Table 1).

**Table 1:** Baseline characteristics of the participants.

	CT + 1.6	CT + 3.2	RT + 1.6	RT + 3.2
**Measure**				
**Anthropometry, body composition, and training experience**				
Age (y)	27 ± 6	25 ± 7	26 ± 6	28 ± 5
Height (cm)	178 ± 5	179 ± 8	180 ± 7	182 ± 6
Body mass (kg)	83.8 ± 10.6	81.6 ± 10.7	82.1 ± 9.1	85.2 ± 10.9
BMI (kg.m^−2^)	26.3 ± 3.4	25.2 ± 3.1	25.1 ± 2.3	25.7 ± 2.9
Training experience (yr)	3.7 ± 2.2	4.6 ± 2.6	3.5 ± 1.7	4.8 ± 2.4
Upper body lean mass (kg)	36.3 ± 4.1	35.8 ± 4	35.7 ± 3.9	39.6 ± 7.3
Lower body lean mass (kg)	20 ± 2.4	20.3 ± 1.9	20.6 ± 2.9	21.6 ± 4.1
**Muscle strength, power and endurance**				
Absolute chest press strength (kg)	97 ± 22	101 ± 19	98 ± 18	108 ± 16
Relative chest press strength (kg. kg BM^−1^)	1.16 ± 0.24	1.25 ± 0.32	1.22 ± 0.31	1.28 ± 0.22
Chest press endurance (rep)	12 ± 2	13 ± 2	11 ± 2	12 ± 2
Absolute leg press strength (kg)	412 ± 77	388 ± 74	390 ± 71	408.7 ± 69.5
Relative leg press strength (kg. kg BM^−1^)	4.97 ± 1.12	4.85 ± 1.23	4.83 ± 1.17	4.87 ± 1.08
Lower body endurance (r)	14.1 ± 2.8	15.3 ± 3.3	14.8 ± 3.1	15.2 ± 2.2
Absolute upper body power (W)	496 ± 62.4	505 ± 92.6	456.9 ± 61.3	509 ± 70.9
Relative upper body power (W. kg BM^−1^)	5.63 ± 0.80	6.32 ± 1.66	5.63 ± 1.07	6.05 ± 1.10
Absolute lower body power (W)	696.2 ± 91.8	738.8 ± 83	695.8 ± 53	752.1 ± 68.8
Relative lower body power (W. kg BM^−1^)	8.38 ± 1.36	9.21 ± 1.81	8.60 ± 1.40	8.96 ± 1.44
Vertical jump (cm)	50.6 ± 4.5	47.8 ± 8.2	43 ± 6.9	44.9 ± 7.1
Pull-up (rep)	11.3 ± 2.8	13.2 ± 3	12.7 ± 2	14.3 ± 4.3

Values are presented as mean ± standard deviation. BMI, body mass index; VO_2max_, maximum rate of oxygen consumption; y, year; cm, centimeter; kg, kilogram; kg.m^−2^, kilogram-meter ^−2^; g, gram; rep, repetition; CT + 1.6, concurrent training + 1.6 g^.^kg^-1.^d^−1^; CT + 3.2, concurrent training + 3.2 g^.^kg^-1.^d^−1^; RT + 1.6, resistance training + 1.6 g^.^kg^-1.^d^−1^; RT + 3.2, resistance training + 3.2 g^.^kg^-1.^d^−1^.

### Lean mass

All four intervention groups demonstrated significant increases in upper [CT + 1.6 = 1.15 kg (95% CI = 0.34 to 1.96; *p* = 0.0026), CT + 3.2 = 1.26 kg (95% CI = 0.45 to 2; *p* = 0.0009), RT + 1.6 = 1.2 kg (95% CI = 0.39 to 2; *p* = 0.0015)], and RT + 3.2 = 1 kg (95% CI = 0.20 to 1.82; *p* = 0.0088) and lower body lean mass [CT + 1.6 = 0.68 kg (95% CI = 0.11 to 1.25; *p* = 0.0120), CT + 3.2 = 0.66 kg (95% CI = 0.09 to 1.22; *p* = 0.0019), RT + 1.6 = 0.82 kg (95% CI = 0.25 to 1.39; *p* = 0.0019)], and RT + 3.2 = 1 kg (95% CI = 0.52 to 1.65; *p* < 0.0001) from baseline to post-test with no differences between groups (*p* > 0.05).

### Muscle strength

All four intervention groups had significant increases in absolute chest press strength [CT + 1.6 = 10.09 kg (95% CI = 6.77 to 13.41; *p* < 0.0001), CT + 3.2 = 10.36 kg (95% CI = 7.048 to 13.68; *p* < 0.0001), RT + 1.6 = 12.55 kg (95% CI = 9.23 to 15.86; *p* < 0.0001)], and RT + 3.2 = 12.91 kg (95% CI = 9.59 to 16.22; *p* < 0.0001), relative chest press strength [CT + 1.6 = 0.10 kg. kg BM^−1^ (95% CI = 0.052 to 0.162; *p* < 0.0001), CT + 3.2 = 0.11 kg. kg BM^−1^ (95% CI = 0.06 to 0.17; *p* < 0.0001), RT + 1.6 = 0.12 kg. kg BM^−1^ (95% CI = 0.06 to 0.17; *p* < 0.0001)], and RT + 3.2 = 0.13 kg. kg BM^−1^ (95% CI = 0.07 to 0.18; *p* < 0.0001), absolute leg press strength [CT + 1.6 = 72.64 kg (95% CI = 44.07 to 101.2; *p* < 0.0001), CT + 3.2 = 74.82 kg (95% CI = 46.26 to 103.4; *p* < 0.0001), RT + 1.6 = 82.36 kg (95% CI = 53.80 to 110.9; *p* < 0.0001)], and RT + 3.2 = 76.82 kg (95% CI = 48.26 to 105.4; *p* < 0.0001), and relative leg press strength [CT + 1.6 = 0.81 kg. kg BM^−1^ (95% CI = 0.47 to 1.15; *p* < 0.0001)], CT + 3.2 = 0.89 kg. kg BM^−1^ (95% CI = 0.55 to 1.23; *p* < 0.0001), RT + 1.6 = 0.87 kg. kg BM^−1^ (95% CI = 0.53 to 1.21; *p* < 0.0001), and RT + 3.2 = 0.78 kg. kg BM^−1^ (95% CI = 0.44 to 1.11; *p* < 0.0001) from baseline to post-test with no differences between groups (*p* > 0.05).

### Muscle endurance

There was no significant change from baseline to post-test for chest and leg press endurance (*p* > 0.05).

### Muscle power

All four intervention groups noted significant increases in absolute upper body power CT + 1.6 = 29.36 w (95% CI = 18.44 to 40.29; *p* < 0.0001), CT + 3.2 = 31.91 w (95% CI = 20.98 to 42.84; *p* < 0.0001), RT + 1.6 = 44.18 w (95% CI = 33.25 to 55.11; *p* < 0.0001), and RT + 3.2 = 49.45 w (95% CI = 38.53 to 60.38; *p* < 0.0001), absolute lower body power CT + 1.6 = 35.82 w (95% CI = 22.27 to 49.37; *p* < 0.0001), CT + 3.2 = 41.91 w (95% CI = 28.36 to 55.46; *p* < 0.0001), RT + 1.6 = 69 w (95% CI = 55.45 to 82.55; *p* < 0.0001), and RT + 3.2 = 65.64 w (95% CI = 52.08 to 79.19; *p* < 0.0001), relative upper body power CT + 1.6 = 0.32 kg. kg BM^−1^ (95% CI = 0.10 to 0.54; *p* = 0.0013), CT + 3.2 = 0.41 kg. kg BM^−1^ (95% CI = 0.19 to 0.62; *p* = 0.0081), RT + 1.6 = 0.41 kg. kg BM^−1^ (95% CI = 0.19 to 0.62; *p* < 0.0001), and RT + 3.2 = 0.46 kg. kg BM^−1^ (95% CI = 0.24 to 0.67; *p* < 0.0001), and relative lower body power CT + 1.6 = 0.33 kg. kg BM^−1^ (95% CI = 0.09 to 0.56; *p* = 0.0030), CT + 3.2 = 0.44 kg. kg BM^−1^ (95% CI = 0.21 to 0.68; *p* < 0.0001), RT + 1.6 = 0.63 kg. kg BM^−1^ (95% CI = 0.40 to 0.87; *p* < 0.0001), and RT + 3.2 = 0.60 kg. kg BM^−1^ (95% CI = 0.36 to 0.83; *p* < 0.0001) from baseline to post-test with no differences between groups (*p* > 0.05) except for absolute upper and lower body power gains. The increases of absolute upper body power in RT + 1.6 were significantly lower than in RT + 3.2 (*p* = 0.044). Also, regarding absolute lower body power, the increases in RT + 3.2 was significantly greater than in CT + 1.6 (*p* = 0.034).

### Muscle performance

All four intervention groups noted significant increases in vertical jump CT + 1.6 = 2.90 cm (95% CI = 0.88 to 4.93; *p* = 0.0022), CT + 3.2 = 4.72 cm (95% CI = 2.70 to 6.75; *p* < 0.0001), RT + 1.6 = 3.54 cm (95% CI = 1.52 to 5.56; *p* = 0.0002), and RT + 3.2 = 3.45 cm (95% CI = 1.43 to 5.47; *p* = 0.0003) and pull-up CT + 1.6 = 3.54 reps (95% CI = 2.05 to 5.04; *p* < 0.0001), CT + 3.2 = 3.81 reps (95% CI = 2.32 to 5.31; *p* < 0.0001), RT + 1.6 = 4.72 reps (95% CI = 3.23 to 6.22; *p* < 0.0001), and RT + 3.2 = 5 reps (95% CI = 3.50 to 6.49; *p* < 0.0001) from baseline to post-test with no differences between groups (*p* > 0.05).

### Associations between changes in lean mass and muscle performance

#### Upper body

**Figure 1:**
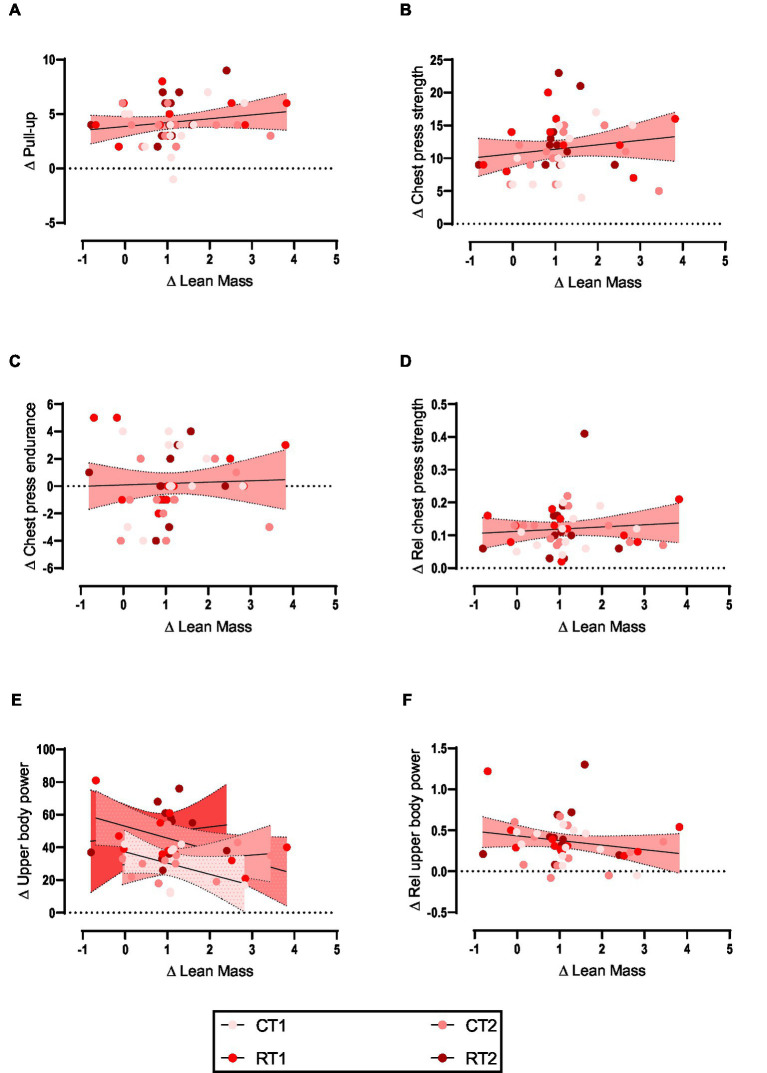
**(A–F)** linear regression (Pearson’s) of Δ (performance) as a function of Δ upper body lean mass (kg). Linear regression is indicated by solid black lines for single models and different lines for others, 95% confidence intervals are indicated by different colors as legends show.

Weak non-significant positive relationships were observed between change in upper body lean mass and changes in pull-up (Figure 1A), chest press strength (Figure 1B), chest press endurance (Figure 1C), and relative chest press strength (Figure 1D) while weak non-significant negative relationship for upper body power (Figure 1E) and relative upper body power (Figure 1F). Data are shown in Table 2 and Figure 1.

**Table 2:** The relationship between the upper body lean mass change and performance.

		Δ Lean Mass for CT + 1.6	Δ Lean Mass for CT + 3.2	Δ Lean Mass for RT + 1.6	Δ Lean Mass for RT + 3.2
Pull-up	*r*	0.270	−0.223	0.298	0.484
	95% CI	−0.39 to 0.74	−0.72 to 0.43	−0.36 to 0.76	−0.16 to 0.84
	*p*	0.421	0.509	0.372	0.130
Chest press strength	*r*	0.535	−0.028	0.127	0.193
	95% CI	−0.09 to 0.85	−0.61 to 0.58	−0.51 to 0.67	−0.45 to 0.71
	*p*	0.089	0.933	0.708	0.568
Chest press endurance	*r*	0.129	0.158	−0.098	0.129
	95% CI	−0.51 to 0.67	−0.48 to 0.69	−0.65 to 0.53	−0.50 to 0.67
	*p*	0.704	0.641	0.773	0.703
Relative chest press strength	*r*	0.364	−0.299	0.130	0.244
	95% CI	−0.30 to 0.79	−0.76 to 0.36	−0.50 to 0.67	−0.41 to 0.73
	*p*	0.270	0.371	0.701	0.468
Upper body power	*r*	−0.480	0.202	−0.607	0.145
	95% CI	−0.83 to 0.16	−0.45 to 0.71	−0.88 to −0.01	−0.49 to 0.68
	*p*	0.134	0.551	0.047	0.669
Relative upper body power	*r*	−0.527	−0.135	−0.416	0.262
	95% CI	−0.85 to 0.10	−0.68 to 0.50	−0.81 to 0.24	−0.40 to 0.74
	*p*	0.095	0.690	0.202	0.435

CT + 1.6, concurrent training + 1.6 g^.^kg^-1.^d^−1^; CT + 3.2, concurrent training + 3.2 g^.^kg^-1.^d^−1^; RT + 1.6, resistance training + 1.6 g^.^kg^-1.^d^−1^; RT + 3.2, resistance training + 3.2 g^.^kg^-1.^d^−1^.

#### Lower body

**Figure 2:**
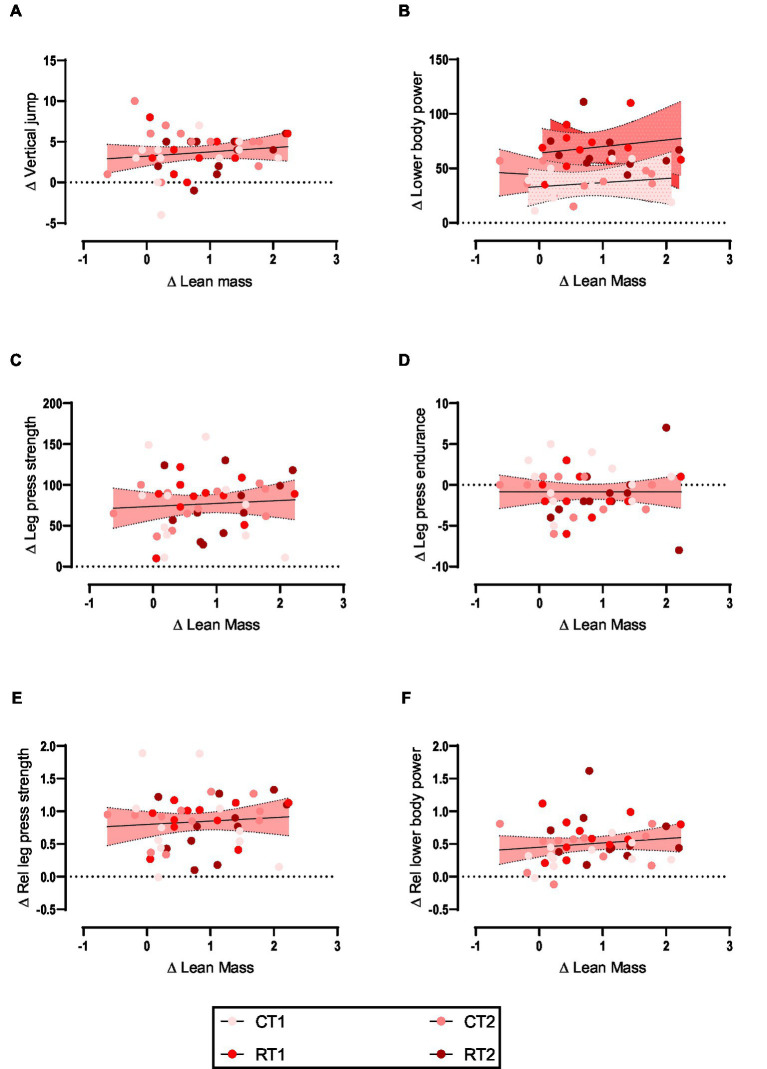
**(A–F)** linear regression (Pearson’s) of Δ (performance) as a function of Δ lean mass (kg). Linear regression is indicated by solid black lines for single models and different lines for others, 95% confidence intervals are indicated by different colors as legends show.

Weak non-significant positive relationships were observed between the change in lower body lean mass with vertical jump (Figure 2A), lower body power (Figure 2B), leg press strength (Figure 2C), leg press endurance (Figure 2D), relative leg press strength (Figure 2E), and relative lower body power (Figure 2F). Data are shown in Table 3 and Figure 2.

**Table 3:** The relationship between the lower body lean mass change and performance.

		Δ Lean Mass for CT + 1.6	Δ Lean Mass for CT + 3.2	Δ Lean Mass for RT + 1.6	Δ Lean Mass for RT + 3.2
Vertical jump	*r*	0.297	−0.075	0.245	0.320
	95% CI	−0.36 to 0.76	−0.64 to 0.54	−0.41 to 0.73	−0.34 to 0.77
	*p*	0.374	0.825	0.467	0.336
Lower body power	*r*	0.179	−0.169	0.201	−0.308
	95% CI	−0.47 to 0.70	−0.69 to 0.47	−0.45 to 0.71	−0.76 to 0.35
	*p*	0.598	0.618	0.552	0.355
Leg press strength	*r*	−0.281	0.332	0.191	0.314
	95% CI	−0.75 to 0.38	−0.33 to 0.77	−0.46 to 0.70	−0.35 to 0.76
	*p*	0.401	0.318	0.572	0.346
Leg press endurance	*r*	−0.035	−0.099	0.102	0.176
	95% CI	−0.62 to 0.57	−0.65 to 0.53	−0.53 to 0.66	−0.47 to 0.70
	*p*	0.917	0.771	0.764	0.603
Relative leg press strength	*r*	−0.264	0.395	0.267	0.400
	95% CI	−0.74 to 0.39	−0.26 to 0.80	−0.39 to 0.74	−0.26 to 0.80
	*p*	0.431	0.228	0.427	0.222
Relative lower body power	*r*	0.357	0.094	0.233	−0.146
	95% CI	−0.30 to 0.78	−0.53 to 0.65	−0.42 to 0.73	−0.68 to 0.49
	*p*	0.280	0.781	0.490	0.666

CT + 1.6, concurrent training + 1.6 g^.^kg^-1.^d^−1^; CT + 3.2, concurrent training + 3.2 g^.^kg^-1.^d^−1^; RT + 1.6, resistance training + 1.6 g^.^kg^-1.^d^−1^; RT + 3.2, resistance training + 3.2 g^.^kg^-1.^d^−1^.

## Discussion

The purpose of this study was to investigate the relationship between changes in lean mass with muscle strength, endurance, and power adaptation responses in resistance-trained males following CT or RT with two different daily high protein doses (1.6 or 3.2 g.kg^−1^.d^−1^). Despite previously observing significant increases in exercise-training induced muscle hypertrophy, strength and selected power responses in both RT and CT modalities, here we found no significant associations between changes in lean mass and performance. The magnitude of non-significant associations was generally similar regardless of exercise training and protein amount, indicating that these factors do not appear to influence associations between lean mass changes and functional adaptation responses.

RT or CT-induced changes in muscle fiber cross-sectional area can be influenced by various factors, including nutrition, genetics, and mechanical factors (49, 50). Such factors can subsequently impact the magnitude of training-induced responses and are also implicated in exercise ‘responder’ and ‘non responder’ paradigm (51). Using resistance-trained participants, our study placed significant focus on the role of training history as an influential variable in the observed results. While this may intuitively lower the possibility of observing correlations between changes in lean mass and strength given there may have been a ‘lower ceiling’ for adaptive responses compared to untrained participants, it also allowed us to utilize a more technically developed and regimented training program to maximize anabolic adaptations. Several studies have compared changes in lean mass and selected measures of muscle performance following diverse exercise protocols. Raymond-Pope et al. (33) observed a positive association between changes in lean mass and strength in NCAA Division I college athletes (33). Others have similarly reported associations between muscle mass and explosive power in young adults (52, 53) while a higher level of muscle strength has also been demonstrated in athletes with higher lean mass (54). In contrast, several studies have not observed any correlations between changes in muscle strength or power and muscle hypertrophy (55–57). The basis with this variability in the literature is likely to be a result of several different including variations in the methods used to conduct tests on subjects, differences in how changes are measured, and variations in the composition of subject groups. For instance, factors such as age, gender, physical condition, and training history can significantly impact study outcomes. Researchers may encounter differences when comparing professional athletes to non-athletes. Another key factor in our study was the inclusion of the CT groups. To our knowledge, this is the first study to examine such correlations in muscle adaptation measures with this training modality. Many individual and team-based sports such as football, rugby and basketball utilize CT as part of their performance as they require a diverse combination of muscle strength, explosive power and aerobic capacity in addition to match-specific movements (e.g., jumping, tackling, changing directions, rapid acceleration, etc.) for successful performance. Thus, investigating the correlation in adaptive responses with CT as we have undertaken in this current study can provide important information as to their specific exercise training variables that may be more beneficial for optimizing outcome measures.

No significant protein-by-group differences were found in associations between lean mass and muscle strength and power adaptation responses in this investigation, demonstrating that alterations occur independently of protein intake. The combination of adequate protein intake and regular physical activity is crucial for enhancing gains in lean mass and muscle strength, but the effects are small (26, 58). A protein intake of 1.6 g^.^kg^-1.^d^−1^ seems adequate to maximize lean mass, muscle strength and power adaptation responses in both RT and CT groups, and aerobic capacity in CT groups, as we demonstrated in our earlier work, except for peak power (35). Notably, recent evidence suggests that there may actually be no upper limit in magnitude and duration of MPS responses to protein ingestion during recovery from exercise *in vivo* in humans (59). Specifically, using a comprehensive quadruple isotope tracer feeding-infusion approach, this study showed that the ingestion of 100 g protein results in a greater and more prolonged (>12 h) anabolic response when compared to the ingestion of 25 g protein. However, collective findings from a number of studies posit that daily protein intakes up to 2.2 g^.^kg^-1.^d^−1^ are adequate for maximizing muscle protein accretion with resistance exercise (60). Moreover, ingestion of 5.5 times the recommended dietary allowance (RDA) of protein (4.4 g.kg^−1^.d^−1^) resulted in similar gains in FFM compared to 1.8 g.kg^−1^.d^−1^ in resistance-trained individuals who otherwise maintain the same training regimen (61). Another study assessed protein requirements during the early stages of training in 12 detrained (for at least 1 year) novice male bodybuilders who received 2.62 g.kg^−1^.d^−1^or 1.35 g.kg^−1^.d^−1^of protein while following an isoenergetic diet for 4 weeks (62). Although the increased protein intake resulted in somewhat larger improvements in certain measurements, the disparities between the conditions were not statistically significant. These results show that during the early stages of reinitiating an RT program, there is no benefit to consuming very high amounts of protein. Our findings thus support a seemingly growing consensus that an optimal daily protein intake for maximizing muscle mass gain with exercise, whether it be RT or now with CT, is typically between 1.6 and 2.2 g.kg^−1^.d^−1^ (60). The human body is capable of assimilating substantial quantities of dietary protein. Nevertheless, the protein translational machinery does not employ all of the constituent amino acids to produce new proteins. Thus, when protein intake exceeds levels of 1.6 and 2.2 g.kg^−1^.d^−1^, MPS becomes saturated. This leads to an increase in the breakdown of amino acids via oxidation and urea formation, resulting in fewer amino acids being available for MPS (60). These explanations may explain as to why 3.2 g.kg^−1^.d^−1^ of protein intake in the present study did not result in further gains in comparison to 1.6 g.kg^−1^.d^−1^. Given that the gains in lean mass did not show significant differences between protein doses, one could argue that the similarity in strength and power adaptation responses is not solely dependent on lean mass. Further investigation is required to examine these associations across various participants (e.g., trained, untrained, etc.) and to get a more profound comprehension of the underlying processes involved.

Another notable finding from the current study was that there we found no significant improvements in endurance adaptations in either of the exercise and high protein interventions. This finding supports findings from a recent systematic review showing long-term protein supplementation to further enhances CT-mediated increases in skeletal muscle mass and strength/power, but not whole-body aerobic capacity (i.e., VO_2max_/peak) (29). Protein intake following ET has been shown to increase myofibrillar protein synthesis (63) and augment the remodeling of muscle and whole-body proteins (64). Such protein remodeling is theorized to be an important aspect of the acute recovery process after exercise that ultimately underpins the adaptations (e.g., greater muscle power, aerobic capacity) that can accrue with ET (64). In addition to the RT component, participants in the CT group in the current study performed stationary cycling that incorporated a mixture of hill simulation rides of varying intensities that was primarily intermittent (i.e., ‘work’ and ‘rest’ periods) in nature. Considering both the selective improvements in muscle strength and power outcomes, and contractile nature of this ET stimulus of the CT program, this finding of no improvements in ET adaptations may likely relate to the ‘specificity of adaptation’. Indeed, one of the central proponents of exercise physiology is the principle of training specificity that proposes an exercise that training responses/adaptations are tightly coupled to the mode, frequency and duration of exercise performed (65). This would imply that training-induced adaptations mostly occur in muscle fibers that have been recruited during exercise regimen, with little or no adaptive changes occurring in untrained musculature. Indeed, ET adaptations may require higher repetitions with lower loads (66). Thus, irrespective of the high protein availability with both diets in the current study, it is likely that the ET component within the CT group (and lack of in the RT group), was not of the appropriate volume, intensity and frequency of exercise sessions to promote ET adaptations. Whether the incorporation of more ET (i.e., high duration and low-to-moderate intensity) within a CT program can significantly improve endurance performances when combined with high protein availability remains an area of future investigation.

A potential limitation of our current work is the small sample size which may restrict the applicability of the findings to a wider population. Additionally, we did not measure protein excretion. Given that the participants consumed a high-protein diet, it would be prudent for future research to include this significant component. Specifically, the study focused on resistance-trained males, making it less relevant for females or individuals with diverse training backgrounds. It also cannot be ruled out that the ‘one off’ nature of 1-RM testing may have resulted in true maximum strength results not being measured (67) although this was relative for all participants. In summary, the relationship between changes in lean mass and muscle strength and power adaptation responses in resistance-trained males generally did not differ according to protein intake (1.6 or 3.2 g^.^kg^-1.^d^−1^) or training mode (RT or CT). The findings demonstrated that, regardless of the protein intake or the type of training, increases in lean mass exhibited weak positive associations with most upper and lower body muscle strength, endurance, and power adaptation response measures. The study proposes that functional enhancements could be associated with neural adaptations rather than muscle hypertrophy, but this assertion warrants careful interpretation. Furthermore, the duration of the study period might influence the observed associations, and more extensive, long-term investigations could yield further insights.

## Data Availability

The raw data supporting the conclusions of this article will be made available by the authors, without undue reservation.

## Footnotes

^1^ www.randomizer.org
