# Supplementary material for: Association between changes in lean mass, muscle strength, endurance, and power following resistance or concurrent training with differing high protein diets in resistance-trained young males
Source: Front Nutr. 2024 Aug 14;11:1439037. doi: 10.3389/fnut.2024.1439037 (PMC11349518; doi:10.3389/fnut.2024.1439037)
Supplement: Supplementary file 1 [file Data_Sheet_1.docx]

| **Supplementary Table 1.** Resistance training program. | | | | |
| --- | --- | --- | --- | --- |
| **Week** | **Intensity (1-repetition maximum [RM])** | **Set** | **Repetition** | **Rest (seconds)** |
| 1 | 75% | 3 | 12 | 45 |
| 2 | 75% | 3 | 12 | 45 |
| 3 | 75% | 3 | 12 | 45 |
| 4 | 75% | 3 | 12 | 45 |
| 5 | 80% | 3 | 10 | 60 |
| 6 | 80% | 3 | 10 | 60 |
| 7 | 80% | 3 | 10 | 60 |
| 8 | 80% | 3 | 10 | 60 |
| 9 | No training - Testing session | ---- | ---- | ---- |
| 10 | 85% | 4 | 8 | 90 |
| 11 | 85% | 4 | 8 | 90 |
| 12 | 85% | 4 | 8 | 90 |
| 13 | 85% | 4 | 8 | 90 |
| 14 | 90% | 4 | 6 | 120 |
| 15 | 90% | 4 | 6 | 120 |
| 16 | 90% | 4 | 6 | 120 |
| 17 | 90% | 4 | 6 | 120 |
| 18 | No training - Testing session | ---- | ---- | ---- |

| **Supplementary** **Table 2A.** Overview of 16-week endurance training program. | | | | | |
| --- | --- | --- | --- | --- | --- |
| **Session Outlines** | | | | | |
| Session Descriptor | Work Interval | Rest Interval | Work Power % MAP | Rest Power % MAP | Repeats |
| Hill Stimulation | 7 min | ----- | 40 | ----- | ----- |
|  | 5 min | ----- | 52.5 | ----- | ----- |
|  | 3 min | ----- | 62.5 | ----- | ----- |
|  | 3 min | ----- | 70 | ----- | ----- |
|  | 5 min | ----- | 25 | ----- | ----- |
|  | 10 Sec | ----- | 40 | ----- | ----- |
|  | 10 Sec | ----- | 50 | ----- | ----- |
|  | 10 Sec | ----- | 60 | ----- | ----- |
|  | 10 Sec | ----- | 70 | ----- | ----- |
|  | 10 Sec | ----- | 80 | ----- | ----- |
|  | 10 Sec | ----- | 90 | ----- | ----- |
|  | 10 Sec | ----- | 100 | ----- | ----- |
|  | 5 min | ----- | 25-40 | ----- | ----- |
| 6 × 2.5 | 2.5 min | 1 min | 70% | 40% | 6 |
| 7 × 2.5 | 2.5 min | 1 min | 70% | 40% | 7 |
| 4 × 4 | 4 min | 1 min | 70% | 40% | 4 |
| 6 × 4 | 4 min | 1 min | 70% | 40% | 6 |
| 7 × 4 | 4 min | 1 min | 70% | 40% | 7 |
| 8 × 4 | 4 min | 1 min | 70% | 40% | 8 |
| HIIT Session A | 10 sec | 50 sec | 100% | 40% | 6 |
|  | 60 sec | 60 sec | 100% | 40% | 1 |
|  | 20 sec | 40 sec | 100% | 40% | 3 |
|  | ----- | 4 min | ----- | 40% | ----- |
| Repeat all parts 2x after 4 min rest | | | | | |
| HIIT Session B | 3 min | 1 min | 70% | 40% | 3 |
|  | 10 sec | 50 sec | 100% | 40% | 6 |
|  | 60 sec | 60 sec | 100% | 40% | 1 |
|  | 20 sec | 40 sec | 100% | 40% | 3 |
|  | - | 4 min | ----- | 40% | ----- |
| Repeat all parts 2x after 4 min rest | | | | | |
| Steady State | 30 min | ----- | 50% | ----- | ----- |

**Abbreviations:** MAP, maximum aerobic power; HIIT, high-intensity interval training.

| **Supplementary** **Table 2B.** The detailed endurance training program. | | | | |
| --- | --- | --- | --- | --- |
|  | **Session** | | | |
| Week | 1 | 2 | 3 | 4 |
| 1 | Hill Simulation | 6 × 2.5 | Hill Simulation | 4 × 4 |
| 2 | 7 × 2.5 | Hill Simulation | 6 × 2.5 | 8 × 4 |
| 3 | 4 × 4 | Hill Simulation | 7 × 2.5 | Hill Simulation |
| 4 | 6 × 4 | Hill Simulation +5% | 6 × 2.5 | 4 × 4 |
| 5 | 6 × 2.5 | Hill Simulation +10% | 7 × 4 | Hill Simulation |
| 6 | Hill Simulation + 15% | Steady State | Hill stimulation | 7 × 4 |
| 7 | Hill Simulation | 8 × 4 | Hill Simulation | HIIT A |
| 8 | 7 × 4 | Hill Simulation | HIIT A | HIIT B |
| 9 | No training - Testing session | ------- | ------- | ------- |
| 10 | 8 × 4 | Hill Simulation | HIIT A | Steady State |
| 11 | Hill Simulation | Steady State | Hill Simulation +5% | 8 × 4 |
| 12 | HIIT B | Hill Simulation +10% | HIIT B | 6 × 4 |
| 13 | HIIT B | Hill Simulation +10% | HIIT B | 7 × 2.5 |
| 14 | 8 × 4 | Steady State | HIIT A | Hill Simulation +10% |
| 15 | Hill Stimulation + 15 % | HIIT A | 7 × 2.5 | HIIT A |
| 16 | HIIT A | Hill Stimulation + 15 % | 4 × 4 | HIIT B |
| 17 | HIIT A | Steady State | 7 × 4 | 6 × 4 |
| 18 | No training - Testing session | ---- | ---- | ---- |

**Abbreviation:** HIIT, high-intensity interval training.

******

**Supplementary Figure 1.** The flow of participant recruitment.
